# Effect of Conductive Material Morphology on Spherical Lithium Iron Phosphate

**DOI:** 10.3390/nano8110904

**Published:** 2018-11-05

**Authors:** Lizhi Wen, Jiachen Sun, Liwei An, Xiaoyan Wang, Xin Ren, Guangchuan Liang

**Affiliations:** 1 Institute of Power Source and Ecomaterials Science, Hebei University of Technology, Tianjin 300130, China; stwenlizhi@163.com (L.W.); starrysjc@126.com (J.S.); an2997097363@126.com (L.A.); wxyhbts@163.com (X.W.); ren18404904993@126.com (X.R.); 2 Automobile & Rail Transportation School, Tianjin Sino-German University of Applied Sciences, Tianjin 300350, China; 3 Key Laboratory of Special Functional Materials for Ecological Environment and Information (Hebei University of Technology), Ministry of Education, Tianjin 300130, China; 4 Key Laboratory for New Type of Functional Materials in Hebei Province, Hebei University of Technology, Tianjin 300130, China

**Keywords:** battery internal resistance, conductive material morphology, spherical lithium iron phosphate, carbon nanotube, graphene

## Abstract

As an integral part of a lithium-ion battery, carbonaceous conductive agents have an important impact on the performance of the battery. Carbon sources (e.g., granular Super-P and KS-15, linear carbon nanotube, layered graphene) with different morphologies were added into the battery as conductive agents, and the effects of their morphologies on the electrochemical performance and processability of spherical lithium iron phosphate were investigated. The results show that the linear carbon nanotube and layered graphene enable conductive agents to efficiently connect to the cathode materials, which contribute to improving the stability of the electrode-slurry and reducing the internal resistance of cells. The batteries using nanotubes and graphene as conductive agents showed weaker battery internal resistance, excellent electrochemical performance and low-temperature dischargeability. The battery using carbon nanotube as the conductive agent had the best overall performance with an internal resistance of 30 mΩ. The battery using a carbon nanotube as the conductive agent exhibited better low-temperature performance, whose discharge capacity at −20 °C can reach 343 mAh, corresponding to 65.0% of that at 25 °C.

## 1. Introduction

As a cathode material for the preparation of lithium-ion batteries, lithium iron phosphates have developed at a high speed and occupy an enormous portion of the world market, having skyrocketed with the development of the new energy automobile market [1,2]. Olivine-type LiFePO_4_ has attracted extensive attention owing to its low cost, high theoretical capacity (170 mAh/g), good cycle performance, excellent thermal stability, environmental friendliness, low self-discharge rate and safety [3,4,5,6].

Despite the above-mentioned advantages, there are still two intrinsic drawbacks limiting its further commercial application. One is the poor rate capability caused by its low electronic conductivity (about 10^−11^ S/cm), and the other being its lower lithium ion diffusion coefficient (about 1.8 × 10^−14^ cm^2^/s) at room temperature [7,8]. Considerable measures have been implemented to overcome these drawbacks, such as doping [9,10], size reduction [11], morphological control and coating [12,13]. Making spherical particles has taken the limelight due to better flowability, which is conducive to higher tap density [14] and excellent electrochemical properties of the battery [15,16]. Nevertheless, the processability of spherical materials is still a barrier to be broken down, requiring more binders to keep particles together (cohesion) and bonding the electrode film to the current collector (adhesion) in the operation of the battery [17]. Meanwhile, battery internal resistance may increase due to the non-conductivity of the adhesive, which hinders the electrical conductivity of batteries. In general, implementing carbon-derived agents promisingly enhances the performance of cathode active materials. The conductive agent is used as a mediator [18] to form a conductive network in the cathode materials, which can reduce the contact resistance of the electrode and improve electron transport rate. Mostly, commercial carbon black, such as Super-P and acetylene black, has been used as a conductive agent in Li-ion batteries [19]. Thus far, carbon nanotubes (CNT) and graphene (G) have also been introduced as a conductive agent instead of carbon black [20]. Because CNT and G possess unique tubular and sheet structures, unique mechanical properties and excellent electronic conductivity, thus increasing the conductivity of the materials and decreasing the electrode polarization, these materials have led to an outstanding rate performance in the battery [21,22]. LiFePO_4_/CNT and LiFePO_4_/G composites [23,24,25,26] have recently attracted investigation and reaped great rewards. However, no attention has been given to the inspection of the effects of conductive agent morphology on the processability and electrochemical performance of batteries. 

Therefore, in this paper, the effects of conductive agent morphology on the processing and electrochemical performance of micron-sized spherical lithium iron phosphate was systematically studied. The chosen conductive agents were traditionally granulated Super-P (Hanyu, Tianjin, China), KS-15 (Maanshan, Sichuan, China), linear CNT (Xianfeng, Suzhou, China) and layered graphene (Xianfeng, Suzhou, China). The evaluation consisted of Scanning Electron Microscope (SEM, FEI, Hong Kong, China), battery internal resistance, cycling and low-temperature performance.

## 2. Experimental Section

### 2.1. Preparation of LiFePO_4_/C Battery

LiFePO_4_/C was synthesized by wet ball-milling, spray drying and carbothermal reduction using glucose as a carbon source. Carbon coated spherical LFP, three different conductive agents and polyvinylidene fluoride (PVDF, AR, Chenguang, Sichuan, China) were used for processing N-methyl-2-pyrrolidone (NMP, AR, Shengda, Shandong)-based cathodes. The conductive agents consisted of traditional additives (Super-P and KS-15 combination, referred to as LFP-SK), carbon nanotube (referred as LFP-CNT) and graphene (referred as LFP-G). Carbon nanotubes and graphene particles were dissolved in NMP at 10% solids content with a stirring device (at the speed of 500 r/min), respectively. For the NMP-based cathodes slurry, PVDF was dissolved in NMP (AR, Shengda, Shandong), and then mixed with conductive agent and LFP, respectively, to form a homogeneous slurry. The slurry was then cast onto an aluminum foil and dried at 120 °C for 12 h under vacuum. The final cathode slurry consisted of 3 wt% PVDF, 2 wt% conductive agent and 95 wt% LFP. The anode electrode was prepared by dispersing 95 wt% graphite, 2 wt% Super-P/KS-15 mixture and 3 wt% carboxylmethyl cellulose (CMC, AR, Jiahang, Henan, China) in distilled water to form a homogeneous slurry. The slurry was cast onto a copper foil and dried at 120 °C for 12 h under vacuum. The double-sided surface density of the cathode was 306 g/m^2^ and the anode was 144 g/m^2^.

The lithium core was made by winding a positive and negative electrode plate using a microporous polypropylene (PP) diaphragm. Subsequently, electrolyte was injected and then packed to obtain the 14,500 cylindrical cells. A total of 1 mol/L hexafluorophosphate (LiPF_6_)/dimethyl carbonate (EC) + dimethyl carbonate (DMC) (1:1, vol) was used as the electrolyte. The capacity balancing of the cathode/anode was 1.05.

### 2.2. Material Characterization

The structures of the LiFePO_4_/C sample were analyzed via X-ray diffraction (XRD, D/max-2500, Rigaku, Tokyo, Japan) using a Cu Kα radiation source (K = 0.15406 nm) over a 2θ range of 10–80° at intervals of 0.02°. The particle morphology and size of the materials were examined using SEM (Nova Nano SEM450, FEI, Houston, TX, USA).

### 2.3. Electrochemical Analysis

The electrode resistance was tested using an electrode resistance tester (GDM-8341, Deji, Suzhou, China). Room-temperature discharge performance tests were performed using the battery test system (LAND CT2001A, Wuhan, China) in the voltage range of 2.5–3.65 V. Low-temperature performance was tested using a YSGDW-150 type high-low temperature test box made in Shanghai. The battery internal resistance was evaluated by a resistance tester (NZY-200, Chaosi, Shenzhen, China) in a half-charged state (State of Charge = 50 %). 

## 3. Results and Discussion

### 3.1. Characterization of LiFePO_4_/C Composite

**Figure 1:**
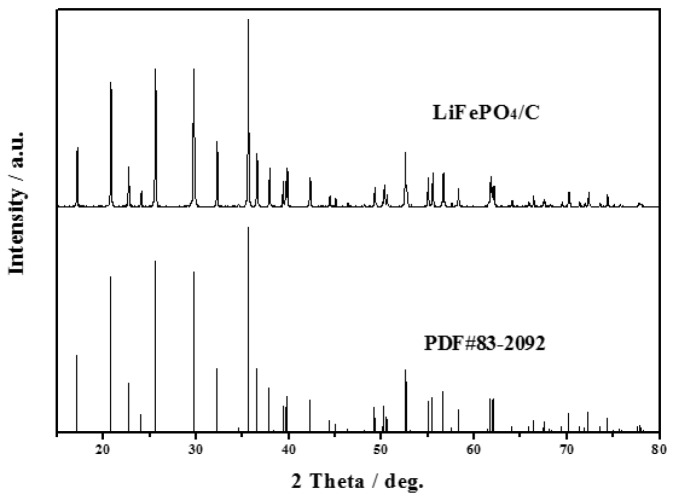
X-ray diffraction (XRD) pattern of LiFePO_4_/C (LFP) sample.

The XRD pattern of LiFePO_4_/C composite is shown in Figure 1. It can be observed in Figure 1 that the sample displays a pure phase with an ordered olivine structure indexed to the orthorhombic Pnmb (PDF#83-2092). No extra diffraction peaks of carbon are found, suggesting that the residual carbon is amorphous and does not affect the structure of LiFePO_4_ [27,28,29,30]. Besides, all the diffraction peaks are narrow and sharp, indicating that the LiFePO_4_/C composite exhibits a high degree of crystallinity. 

### 3.2. Processability of Electrodes

#### 3.2.1. Morphology

**Figure 2:**
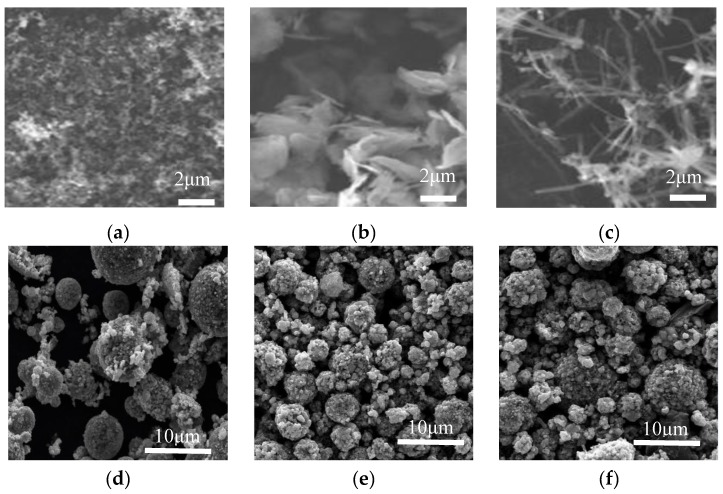
SEM of conductive agents and electrodes with different conductive materials: the surface morphologies of Super-P and KS-15 (**a**); the surface morphologies of graphene (**b**); the surface morphologies of carbon nanotube (**c**); the electrodes image of Super-P and KS-15 (**d**); the electrodes image of graphene (**e**); the electrodes image of carbon nanotubes (**f**).

**Figure 3:**
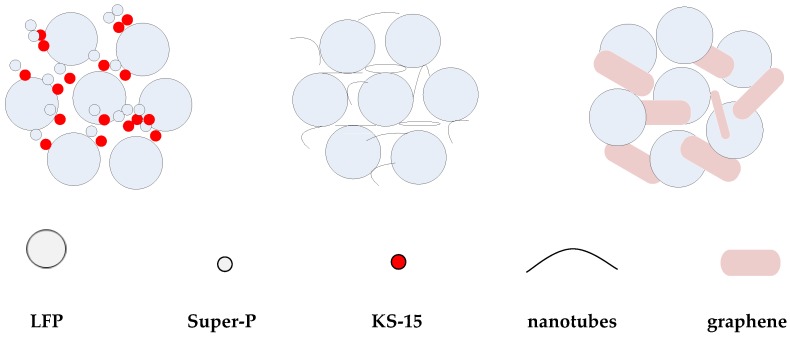
The contact model of electroactive materials and conductive agents.

Figure 2 shows SEM images of the conductive agents and electrodes with different conductive materials. Figure 2a–c are the surface morphologies of conductive agents Super-P and KS-15, G and CNT, and Figure 2d–f are the surface morphologies of electrodes with these materials. In Figure 2a, agglomerates composed of fine carbon particles are shown. In Figure 2b, aggregated graphene flakes with a length ~4 μm can be observed. CNTs are linear in form and entangled, as shown in Figure 2c. These conductive agents display different morphologies, which may influence the conductive network. According to this figure, LFP particles are standard micron-sized secondary spherical particles formed by nano-sized primary particles. As shown in Figure 2d, the smaller particles of LFP can fill in the space between larger ones, which may contribute to high tap density. The conductive agents Super-P and KS-15 fill the gaps between LFP particles; meanwhile, the surfaces of some LFP particles are partially covered with conductive carbon, which could reduce the lithium ion diffusion path. As shown in Figure 2, the CNT can form a conductive network to connect the active materials, thus immensely reducing the contact resistance between particles. The conductive agent G can link the particles together in a point-surface way, to some extent, due to its sheet structure. Moreover, considering the morphological differences between the conductive agents, the use of CNTs and G rather than Super-P/KS-15 seems to be a better choice for forming a well-connected conductive network among the cathode materials. Figure 3 shows the model of the indirect contact of particles. The conductive agent is distributed in the spaces between LFP particles, which can attach to the surfaces of particles to enhance the conductivity between them. The CNTs, which can form a kind of bridge in a way that produces a conductive network, are wrapped around the surfaces of particles and can greatly reduce the contact internal resistance between particles. G can be dispersed in a kind of encapsulating manner, surrounding the surfaces of particles to make particles tightly connected.

#### 3.2.2. Stability of Solid Content

The form of the slurry is the dispersion of solid particles in NMP, and there are attractive and repulsive forces between small particles. The secondary particle agglomeration can be formed through attraction when the attraction force is greater than the repulsive force, this having a great impact on the subsequent coating operation. CNT and G are macromolecular materials with higher specific surface areas and nano-level particles than traditional conductive agent, for which the dispersion is more difficult than traditional conductive agent. Therefore, the determination of slurry stability is necessary. Non-Newtonian fluid is a peculiarity of the cathode material slurry. Sedimentation occurs when the slurry is in a stationary state, and the variation of the slurry solid content at different times is used to characterize the stability of the slurry. The solid content is measured by removing the slurry from the same position in the beaker at different times and then coating the slurry on aluminum foil, determining the weight before and after drying to calculate the solid content (usually expressed as a percentage). The formula is as follows:

*W* = (*m*_2_ − *m*_0_)/(*m*_1_ − *m*_0_) × 100% (1)

Among:

*m*_0_—the weight of the foil
*m*_1_—the weight of pulp with foil before drying
*m*_2_—the weight of pulp with foil after drying

**Figure 4:**
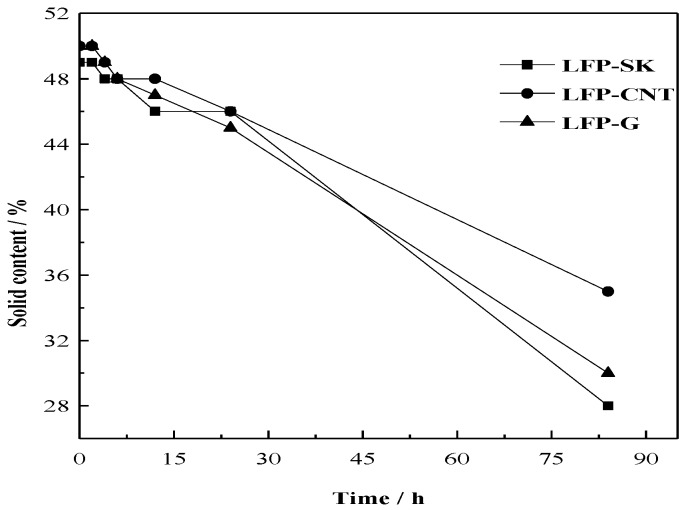
Changes in solid contents over time.

In general, the slurry viscosity should be adjusted to between the range of 8000–10,000 mPa·s. The rheological behavior of slurry was controlled around 8200 mPa·s. Figure 4 shows the change of solid content over time in a stationary state with the conventional conductive agent (LFP-SK), carbon nanotube (LFP-C) and graphene (LFP-G). It can be seen from the figure that, on the whole, the solid content of the three samples decreases with time. In the first 5 h, the slurry is in a stable state, and the stability decreases gradually over time. The retention rates of the solid content after 84 h are 57%, 70% and 60%, respectively, which indicates that the stability of the slurries with added CNT and G are better than that with added traditional Super-P and KS-15. This means that the uniform dispersion of a slurry can be achieved by adding CNT and G during the slurry making process. The liner and sheet structure of CNT and G can improve the slurry stability to a certain extent.

#### 3.2.3. Cohesiveness Properties

The cohesiveness property between a material and aluminum foil is a very important parameter that directly affects the processability of a battery material. The adhesion test method is as follows: the tape is first attached to the pole piece surface and then torn off at a 90° angle to see how much material is torn off by the tape. If most of the coating is left on the aluminum foil, either the adhesion is good, or the adhesive is poor.

**Figure 5:**
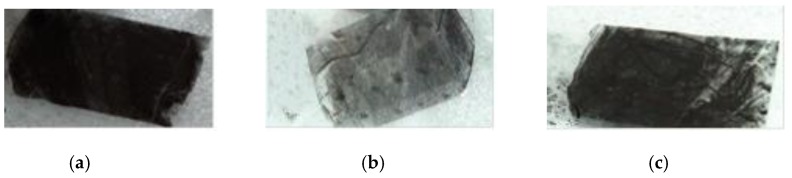
Cohesiveness of different electrodes: LFP-SK (**a**); LFP-CNT (**b**); LFP-G (**c**).

Figure 5a–c shows the pieces of tape pulled from the electrodes with LFP-SK, LFP-CNT and LFP-G, respectively. The figure shows that the most material was detached from LFP-SK electrode, followed by LFP-G. In other words, the electrode with LFP-C as a conductive agent has the best adhesion. That is to say, CNT and G, as conductive agents can improve the cohesiveness of the electrode due to its own unique network structure connecting the cathode active materials. Because the conductive agents Super-P and KS-15 form as particles, and the active material lithium iron phosphate is also comprised of spherical particles, they therefore possess point-point contact. The conductive agents Super-P and KS-15 only accumulate in the gaps that surround the active material. However, the CNT has a linear structure that comes into contact with the active material in a dotted-line model [31], forming a network structure at the surface of active material, keeping the active material tightly together. G is a layered structure in a point-surface contact with active material, which can increase the contact area and improve the stripping effect of the pole pieces. Therefore, the addition of CNT and G as conductive agents can significantly improve the adhesion of the pole piece.

#### 3.2.4. Electrode and Battery Resistance

Electronic conduction is a straightforward representation of electrode resistance, which affects the internal resistance of batteries. The electrode and battery resistances of the samples are shown in Table 1. It can be seen from this table that electrode resistance is associated with battery internal resistance, but not in liner relation. The use of LFP-CNT and LFP-G as conducting agents can reduce the electrode resistance and battery internal resistance, owing to the fact that the high conductivity of CNT and G can reduce the contact resistance of the materials, thus greatly decreasing the internal resistance of the batteries. Given similar proportions, CNT is superior to the other additives regardless of processability or electrochemical performance, while the battery using LFP-CNT has the smallest internal resistance of 30 mΩ. 

### 3.3. Electrochemical Performance

**Figure 6:**
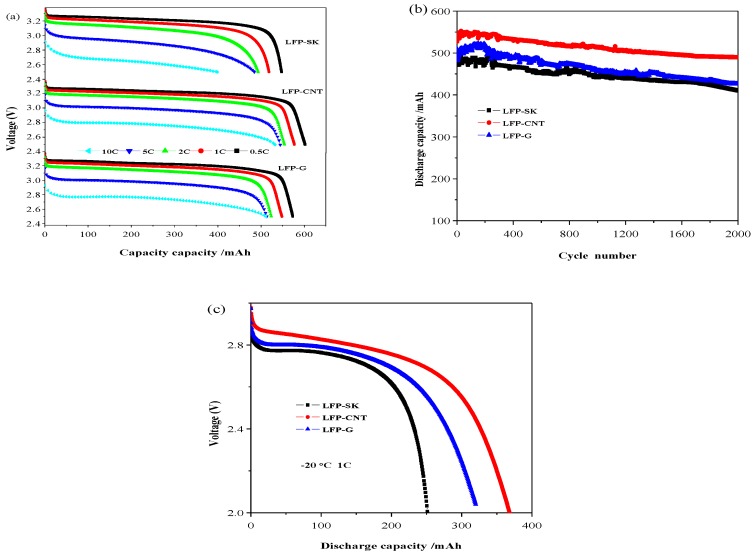
Electrochemical performance of 14,500 batteries with different conductive agents: discharge performance (**a**); cycling performance (**b**); discharge performance at −20 °C at a discharge rate of 1C (**c**).

The electrochemical performance test was carried out using 14,500 batteries with LFP-SK, LFP-CNT and LFP-G. The obtained results are shown in Figure 6. The discharge curves of the batteries with different conductive agents at different discharge rates (0.5 C-1C-2C-5C-10 C) are shown in Figure 6a. For the battery with LFP-SK as the cathode, the discharge capacities are 547, 518, 492, 484 and 398 mAh at 25 °C at 0.5 C, 1C, 2C, 5C, 10 C rates, respectively, and those of LFP-CNT and LFP-G are 602, 577, 554, 544, 532 mAh and 573, 548, 523, 513, 511 mAh. Therefore, the rate performance of LFP-CNT battery is the best, especially at a high rate. Figure 6b shows the cycling performance of the 14,500 full batteries with LFP-SK, LFP-CNT and LFP-G at 1C. According to the figure, the initial discharge capacity of LFP-CNT sample is 539.0 mAh. After 2000 cycles, the discharge capacity remains 493.7 mAh, with a capacity retention rate of 91.6%. While the capacity retention rate of the LFP-SK sample is 84.3%, the capacity retention rate of LFP-G sample is closer to 91.0%. Therefore, the cyclic stability of the LFP-CNT sample is the best. The experimental results prove that the discharge performance of the cylindrical cells can be improved by adding carbon nanotubes and graphene, especially the high rate discharge performance, though the effect of graphene is lower than that of carbon nanotubes. This can be explained as follows. Firstly, carbon nanotubes are one-dimensional linear structures connecting the active material in a way similar to bridging, thus forming a good network structure around the surfaces of the active material particles by winding and cladding. This network structure not only increases the lithium ion diffusion channel, but also increases the contact between the electrode particles to a certain extent, thus improving the stripping effect between particles due to volume changes in the charge-discharge process. Graphene is a layered structure, which can effectively increase the contact area with the active material particles and the physical contact between the particles in the charge-discharge process, thereby increasing the lithium ion diffusion rate, giving the electrode material a better rate and cycling performance.

Low-temperature discharge performance of the materials is shown in Figure 6c. Before the low temperature battery test, the battery was charged and discharged two times at a current density of 500 mA at room temperature. The discharge capacities of LFP-SK, LFP-CNT and LFP-G at −20 °C at 1 C are 252, 374 and 320 mAh, respectively, corresponding to 48%, 65%, and 58% of that at 25 °C. Moreover, the discharge capacity of LFP-SK decreases rapidly. The battery using LFP-CNT as an electrode has a higher discharge capacity at −20 °C, the same as that using LFP-G. That is to say, the batteries using CNT and G, as conductive agents, show better low-temperature performance than that using traditional Super-P and KS-15.

## 4. Conclusions

Three morphologically different conductive agents were added into the slurry, and the 14,500 cylindrical batteries were successfully fabricated by coating and winding. Through a series of physical and electrochemical properties tests, the results show that in terms of the spherical active materials, linear carbon nanotubes and layered graphene have better comprehensive performances, including processing and electrochemical performance, compared with the traditional granular conductive agents Super-P and KS-15. The slurry stability and electrode adhesion are improved, and the battery internal resistance is reduced. Additionally, the battery exhibits excellent circulation and low-temperature performance, owing to the unique connectivity between particles allowing the lithium ion diffusion path to decrease; therefore, the battery has less resistance and better rate performance. The performance of LFP-CNT is more apparent: the solid content change is smaller in a steady state, the pole piece has a better cohesiveness, and the capacity retention rate is 65% at 1 C and −20 °C. That means carbon nanotubes and graphene, with their special structures and high conductive rate, can replace traditional conductive agents to improve the electrochemical performance of the batteries. 

## Figures and Tables

**Table 1:** Electrode and battery resistance.

Samples	Electrode Resistance (Ω)	Battery Internal Resistance (mΩ)
LFP-SK	60	42
LFP-CNT	46	30
LFP-G	43	35
